# The Relationship between Self-Awareness of Attentional Status, Behavioral Performance and Oscillatory Brain Rhythms

**DOI:** 10.1371/journal.pone.0074962

**Published:** 2013-09-17

**Authors:** Noriko Yamagishi, Stephen J. Anderson

**Affiliations:** 1 Department of Cognitive Neuroscience, ATR Cognitive Mechanisms Laboratories, Kyoto, Japan; 2 Japan Science and Technology Agency, PRESTO, Saitama, Japan; 3 Brain Networks and Communication Laboratory, Center for Information and Neural Networks, National Institute of Information and Communications Technology, Osaka, Japan; 4 Neurosciences, School of Life and Health Sciences, Aston University, Birmingham, United Kingdom; University of British Columbia, Canada

## Abstract

High-level cognitive factors, including self-awareness, are believed to play an important role in human visual perception. The principal aim of this study was to determine whether oscillatory brain rhythms play a role in the neural processes involved in self-monitoring attentional status. To do so we measured cortical activity using magnetoencephalography (MEG) and functional magnetic resonance imaging (fMRI) while participants were asked to self-monitor their internal status, only initiating the presentation of a stimulus when they perceived their attentional focus to be maximal. We employed a hierarchical Bayesian method that uses fMRI results as soft-constrained spatial information to solve the MEG inverse problem, allowing us to estimate cortical currents in the order of millimeters and milliseconds. Our results show that, during self-monitoring of internal status, there was a sustained decrease in power within the 7-13 Hz (alpha) range in the rostral cingulate motor area (rCMA) on the human medial wall, beginning approximately 430 msec after the trial start (p < 0.05, FDR corrected). We also show that gamma-band power (41-47 Hz) within this area was positively correlated with task performance from 40–640 msec after the trial start (r = 0.71, p < 0.05). We conclude: (1) the rCMA is involved in processes governing self-monitoring of internal status; and (2) the qualitative differences between alpha and gamma activity are reflective of their different roles in self-monitoring internal states. We suggest that alpha suppression may reflect a strengthening of top-down interareal connections, while a positive correlation between gamma activity and task performance indicates that gamma may play an important role in guiding visuomotor behavior.

## Introduction

Over the past decade, numerous experimental paradigms have been employed to ascertain the roles played by higher-level cognitive factors in primate vision, including attentional processes and self-awareness. Several key findings follow. Physiological studies have shown that the lateral intraparietal area, frontal eye field and superior colliculus are involved in guiding visual attention [1], while the neural correlates of focal attentive processes can be observed in areas V1, V2 and V4 under conditions that require stimulus feature analysis and selective spatial processing within a field of competing stimuli [2,3]. Similarly, human neuroimaging studies employing functional magnetic resonance imaging (fMRI) show that attentional activation within primary visual areas is task dependent [4]. Electro- (EEG) and magneto-encephalographic (MEG) studies on human vision show that attentional modulation either before or after stimulus presentation affects the amplitude of occipital alpha rhythms [5,6]. Recent multiunit recordings on primate have also demonstrated that attentional modulation affects alpha (and gamma) rhythms in early visual areas [7]. Finally, several behavioral studies on humans have demonstrated that attention enhances visual sensitivity and performance [8,9].

Evidence is also emerging, through behavioral studies, that humans can self-monitor their attentional status for the purpose of enhancing visual performance [10]. Smith et al. [11] reported that mirrored self-faces were more rapidly recognized and more strongly identified when self-awareness was internally directed compared with socially directed, whereas the opposite was true for the perception of un-mirrored self-faces. They concluded that self-awareness has stimulus-specific effects on visual perception, and that states of self-awareness might generally provide internal cues to selectively enhance behaviourally relevant visual signals. Yamagishi et al. [10] used an orientation discrimination task to determine whether attentional processes can be internally monitored for the purpose of enhancing performance. In their experiments observers had the freedom to delay target presentation – by any amount required – until they judged their attentional focus to be complete. In doing so, Yamagishi et al. reported that observers were able to improve significantly their orientation discrimination performance. They concluded that attentional mechanisms can be self-monitored for the purpose of enhancing human decision making processes.

While the neural mechanisms underlying visual attention have – at least in part – been revealed by physiological and neuroimaging studies, the neurophysiological basis underlying self-monitoring processes remains unknown, although a recent meta-analysis of fMRI data suggested that the anterior cingulate may play a role in processing ‘self-specific’ stimuli [12]. In this study, we used a combination of magnetoencephalographic and functional magnetic resonance imaging to examine the neural processes underlying our ability to self-monitor attentional status. Given the purported roles of both low- and high-frequency oscillatory brain rhythms in defining attentional status, our principal aim in this study was to determine whether such rhythms also play a role in the self-monitoring of attentional status. 

## Materials and Methods

### Participants

Ten right-handed individuals (1 female) between 20 and 27 years of age (mean 23.6) with normal or corrected-to-normal vision participated in all behavioral, fMRI and MEG experiments. All individuals were screened for neurological and/or psychiatric abnormalities, and all were paid for their participation.

### Ethics Statement

All participants gave written informed consent for the experimental procedures, approved by the ATR Human Subject Review Committee, and the experiments were run in accordance with the Helsinki Declaration.

### Stimuli and task

All stimuli were generated using a VSG2/5 graphics board (Cambridge Research Systems, UK). For the MEG experiment, images were projected (DLA-G150CL projector, Victor Company of Japan, Ltd, Japan) from outside a magnetically shielded room onto a semi-translucent screen inside the room. For the fMRI experiment, images were projected (DLA-G150CL projector, Victor Company of Japan, Ltd, Japan) from outside a magnetically shielded room onto a semi-translucent screen and reflected onto a mirror attached to a head coil on the MRI scanner. The mean luminance (40 cd/m^2^) and colour (CIE coordinates: x=0.31, y=0.32) of the stimuli were matched to that of the surround.

The target stimulus was a Gaussian-modulated (σ=0.5°) sinusoid of two cycles/degree periodicity, occupying 3° x 3° of visual angle at a viewing distance of 36 cm (for MEG experiment) or 96 cm (for fMRI experiment). Its orientation was rotated from the horizontal meridian by 5° (clockwise or anti-clockwise with equal probability), and its contrast was 1.4 times each participant’s threshold, which was pre-defined using a 3-up 1-down stair-case method. Two distracter targets were presented either side of the target stimulus. Their size and spatial structure were same as the target but their orientation was randomly chosen from ±90° from the horizontal, and their contrast was fixed at 50%. The distracters and the target stimulus were aligned horizontally and separated by 3° (center-to-center spacing). The center of the target stimulus was 7° directly below fixation.

### Procedure

**Figure 1:**
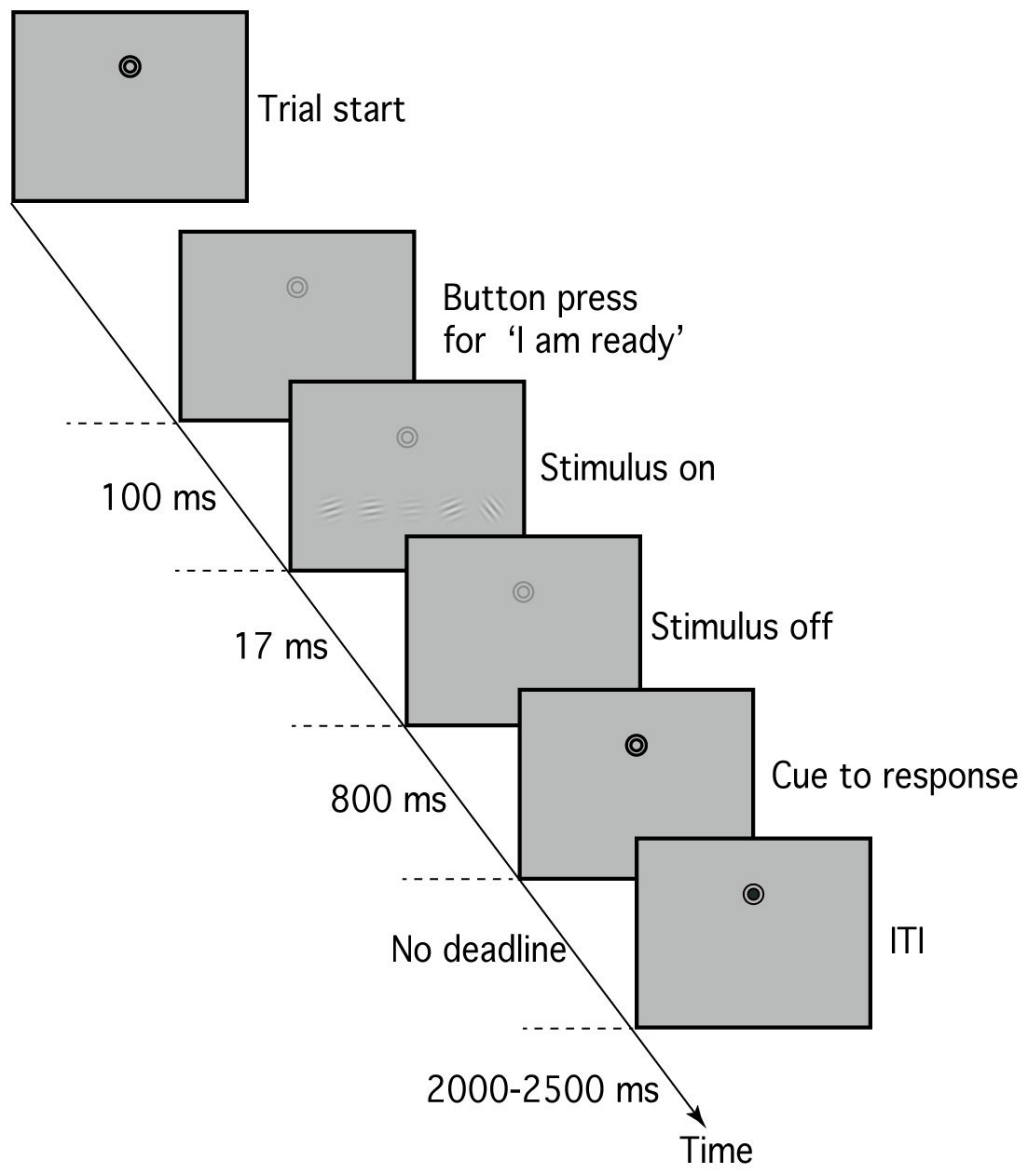
Trial sequence for the observer-control condition. Participants were asked to monitor their internal status and, when they judged their attention to be maximal, depress a button to initiate presenting stimuli. The passive viewing condition followed the same procedure except that the time before the stimulus onset was controlled by a computer selecting from the recorded latencies in the previous block of the observer control condition.

There were two experimental conditions: (A) observer-control condition, and (B) passive viewing condition. In both conditions, each trial began with the presentation of a black bull’s-eye fixation target (two concentric open circles with radii of 0.2° and 0.4°). Participants were instructed to maintain fixation during each trial and avoid blinking except during the inter-trial interval (ITI). In the observer-control condition, participants were asked to monitor their internal status and, when they judged their attention to be maximal, depress a control-box button to initiate stimulus presentation (Figure 1). On depression of the button, the fixation colour changed from black to gray. One hundred milliseconds later, the target stimulus and the distracters were presented for one frame (17 msec). The participant’s task was to judge whether the target grating was clockwise (right-hand button press) or anti-clockwise (left-hand button press) tilted. To prompt a response, the fixation colour was changed 800 msec after stimulus offset. No response deadline was imposed. An ITI of 2000-2500 msec followed the response. The passive viewing condition followed the same general procedure except that: (i) the duration between the start of the trial and stimulus onset was controlled by a computer – the length of this pre-stimulus period was selected pseudo-randomly from the distribution of latencies recorded in the previous block of observer-control trials; and (ii) no response was required – the time period prior to the ITI was fixed at 1000 msec.

There was a baseline period of 1000 msec prior to the beginning of each trial in the MEG experiments.

### MEG acquisition

Magnetic responses were recorded using a 210-channel whole-head bio-magnetic imaging system (PQ1400RM; Yokogawa Electric Co., Japan). The responses were continuously sampled at 1 kHz during each session. Simultaneous recording of the electro-oculogram (EOG) was used to detect and discard epochs with eye movement or blink artifacts. Each observer participated in four sessions. Each session contained two blocks of experiments: one for the observer-control condition, and another for the passive viewing condition. Each block contained 40 trials. A total number of 160 trials recorded for each condition. Before the MEG experiment, each participant’s face and head shape were scanned using a hand-held laser scanner and a stylus marker (FastSCAN; Polhemus, USA), completed for later use in co-registration of the MEG functional data with the MRI structural data. The observer’s head position in the MEG system was measured using a standard facial arrangement of calibration coils. Electromagnetic calibration of the coil position was conducted before and after each session.

### fMRI acquisition

A 1.5-tesla MRI scanner (Shimadzu-Marconi Magnex Eclipse) was used to acquire both structural T1-weighted images (TR = 20 ms, echo time [TE] = 2.3 ms, flip angle = 40°, matrix = 256x 256, field of view [FoV] = 256 mm, thickness = 1 mm, slice gap = 0 mm) and T2* -weighted echo planar images (TR = 2.5 s, TE = 49 ms, flip angle = 80°, matrix = 64x 64, FoV = > 192 mm, thickness = 5 mm, slice gap = 0 mm, 25 slices), showing BOLD contrasts. Each observer participated in eight experimental sessions. Each session contained six blocks of experiments. Blocks of the (1) observer-control condition, (2) passive viewing condition and (3) rest condition were repeated twice in each session. Note that in the rest block condition, only a fixation point was present on-screen, presented for the same length of time as in the previous passive viewing condition. Each experimental block contained six trials. A total of 96 trials of data were recorded for each experimental condition. The total scan number for each session varied between 108 and 160, depending on the required time for participants to judge they were ready for the task.

### fMRI analysis

Functional imaging data were analyzed using SPM5 (Wellcome Trust Centre for Neuroimaging, London, UK; http://www.fil.ion.ucl.ac.uk/spm). We discarded the first six volumes of images in each session to allow for T1 equilibration, and then spatially aligned the data to the first remaining volume. The data were spatially normalized to the Montreal Neurological Institute (MNI; Montreal, Quebec, Canada) reference brain, and spatially smoothed with a Gaussian kernel (8 mm, full-width at half-maximum).

Statistical analyses were performed for each participant. Boxcar functions were modeled for each condition, including the rest condition. They were convolved with the canonical hemodynamic response function in SPM5 to yield regressors in a general linear model. A parameter was estimated for each regressor using a least-squares method. T-statistics were used for comparison between the estimated parameters (observer-control versus rest condition, and passive viewing versus rest condition) to yield a t-value for each voxel. Because both Sato et al. [13] and Yoshioka et al. [14] have demonstrated that the hierarchical Bayesian method is robust with respect to false-positive prior information in fMRI signals, we used a threshold of p<0.001 (uncorrected for multiple comparisons), as previous studies [14,15] have done for the analysis of hierarchical Bayesian estimation for MEG [13]. The statistical parametric maps were combined using a logical OR operation, and used as prior information for the estimation of MEG source currents. To specify regions of interest (ROI) for self-monitoring of attentional status, each individual’s results (observer-control versus passive viewing condition) were combined across participants (i.e. random effect, with n = 10). Statistical significance was evaluated with a height threshold of p < 0.001 uncorrected and extended threshold equal to 30.

### MEG preprocessing

Magnetic response signals were high-pass filtered at 1 Hz, and used to generate two data sets for each participant: (1) data time-locked to the start of each trial, and (2) data time-locked to the depression of the control-box button for the observer-control condition. A baseline measure was computed from the magnetic signals recorded during the 300 msec prior to the start of each trial. Eye movement artifact rejection was performed offline by removing epochs with an EOG peak amplitude exceeding 100 μv.

### Cortical current estimation using the hierarchical Bayesian method

A polygon cerebral cortex model was constructed for each participant based on MR structural images by using Brain Voyager software (Brain Innovation, Maastricht, Netherlands). The total number of vertex points for each participant was 27221 ± 368 (mean ± standard error [SE]). A cortical dipole model was constructed from the polygon model by assuming the existence of a current dipole at each vertex point. The dipoles were equidistantly distributed and the dipole current directions were assumed to be perpendicular to the cortical surface.

For each participant, we estimated the cortical current of each dipole using a hierarchical Bayesian method [13,14]. This method estimates cortical activity from MEG sensor signals in a distributed source model in which cortical current is modeled by a number of dipoles with fixed position and orientation. In this method, the variance of the cortical current at each location is considered an unknown parameter, and estimated from the observed MEG data and prior information derived from fMRI [16].

For each trial, the duration from the start of a trial to the subsequent button press by a participant – signaling they are ready for the stimulus to be presented – necessarily varied from trial to trial. Trials were eliminated if the EOG peak amplitude exceeded 100 μv (rejection rate = 12.7% ±3.0%). To estimate cortical currents, we used the MEG data with the longest trial duration because our method of analyses [13] requires that all trials have the same length.

Assuming the pattern of cortical activity varies with the participant’s internal state, we calculated an inverse filter for each 100 msec time window, with a 50 msec overlap in each trial. The cortical current was then estimated with the MEG data using the filter at each time window. In the overlapping period, the cortical currents were averaged between successive time windows. Note that estimated currents cannot be influenced by data that fall outside the 100 msec time window [13].

Event-related spectral perturbation (ERSP) plots [17] were generated for each condition and participant using the single-trial estimated current for each ROI – each pixel on the ERSP plot was submitted to statistical analysis. The correlations between task performance and ERSP values were also tested by determining a correlation coefficient between task performance (accuracy) and ERSP values at each frequency and at each time point for each participant.

## Results

### Behavioral results

Preliminary behavioral measures were completed to determine whether, under the experimental conditions employed in this study, self-monitoring of attentional status did improve visual performance, as reported by Yamagishi et al. [10]. We can confirm that performance in the orientation judgment task was significantly affected by experimental condition: performance was greatest for condition A, where participants self monitored their attentional status (mean accuracy: Condition A, 81.3%; Condition B, 67.8%; t-test p < 0.01).

Averaged across participants, performance for the bar-orientation judgment task was 77.6 ± 3.2% correct [mean ± SE] in the MEG experiment, and 79.2 ± 3.3% correct [mean ± SE] in the fMRI experiment. There was no statistical difference for this task between the MEG and fMRI experiments (t-test p > 0.5), confirming anecdotal reports by observers that the levels of task difficulty appeared equal.

**Figure 2:**
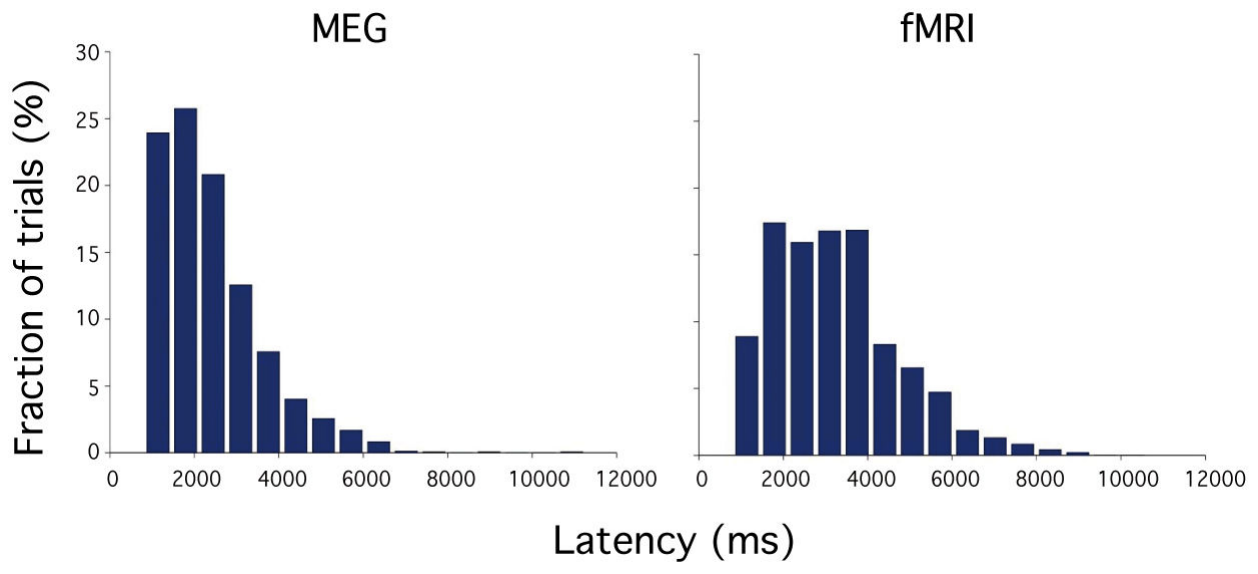
Group data (n = 10) showing latency distribution for signaling maximal attentional status in the MEG experiment (left panel) and in the fMRI experiment (right panel).

The elapsed time required for participants to judge that their attentional focus was maximal varied from trial to trial (Figure 2), in a similar fashion to that found previously in our behavioral work [10]. The elapsed time ranged from 812 msec to 11.3 seconds (median 2.17 s) in the MEG experiment, and from 828 msec to 9.2 seconds (median 3.06 s) in the fMRI experiment.

### Regions of Interest (ROI)

**Figure 3:**
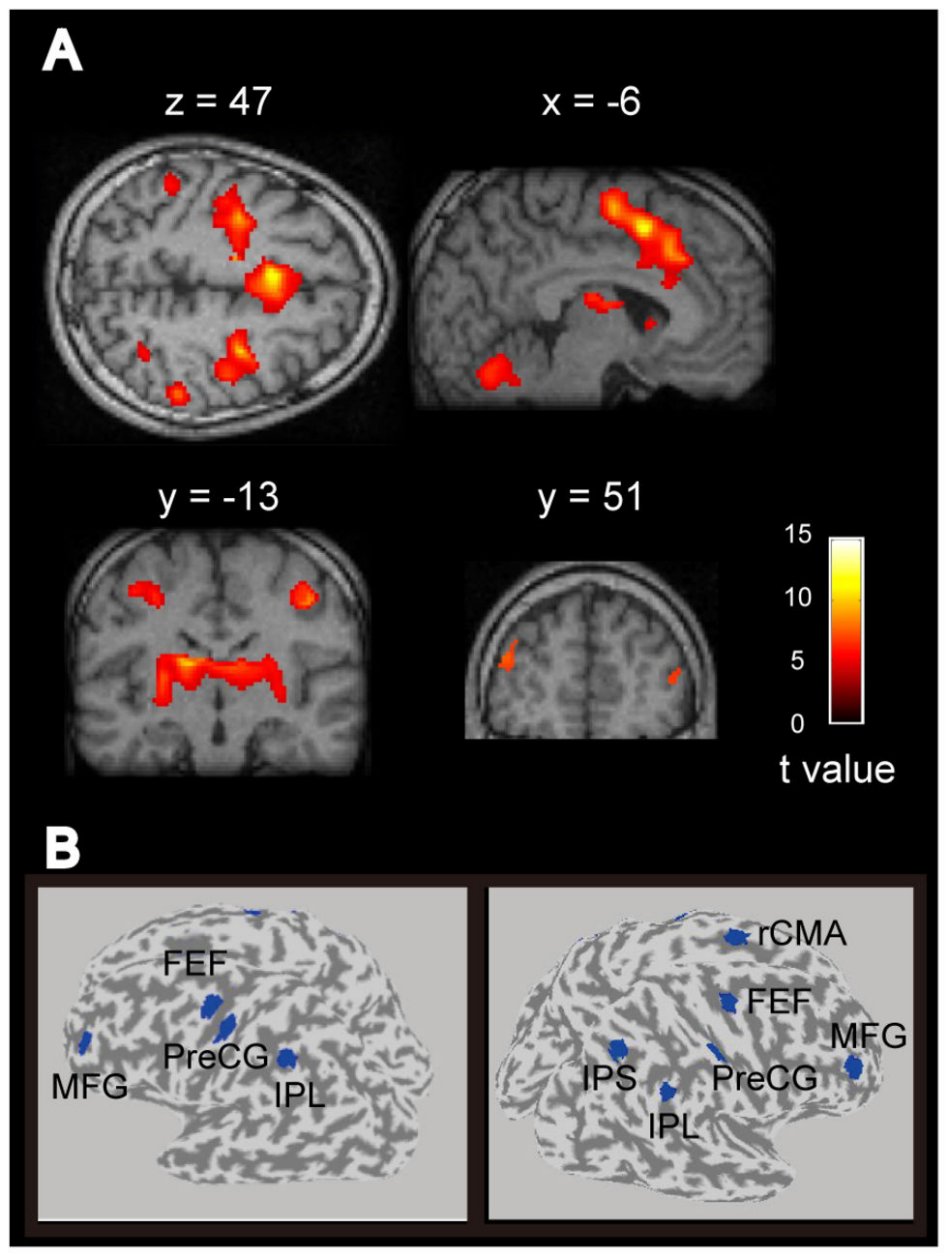
Self-monitoring related activity in fMRI experiments and ROI specification. **A**, Brain areas activated more during the observer-control condition than the passive viewing condition (P < 0.001, uncorrected; extended threshold of 30 voxels). **B**, Ten ROIs were defined by specifying a circular region with a 6-mm radius centered at the coordinates shown in Table 1. Abbreviations are the same as in Table 1.

ROIs were determined for each participant by contrasting the fMRI results for the observer-control and passive viewing conditions (see Methods). Figure 3A shows the group (n = 10) results (see also Table 1; p < 0.001 uncorrected, extended threshold = 30), and indicates which cortical regions were activated more during the observer-control condition than the passive viewing condition. A total of ten areas were identified, all within the frontal and parietal cortex (Table 1). MNI coordinates were converted into Talairach coordinates using a linear transformation matrix. Ten ROIs [left middle frontal gyrus (MFG), right MFG, left frontal eye field (FEF), right FEF, rostral cingulate motor area (rCMA), left precentral gyrus (preCG), right preCG, left inferior parietal lobe (IPL), right IPL, right intraparietal sulcus (IPS)] were then determined for each participant by specifying a circular region with a 6-mm radius centered at the coordinates shown in Table 1 (Figure 3B). Each ROI contains 16 ± 1 dipoles (mean ± SE). A representative dipole for each ROI was determined by averaging the estimated cortical current amplitudes for each dipole – over the time period from the trial start to depression of the control-box button – and then selecting the dipole with the largest value.

**Table 1:** Event-related activations to cues for self-monitoring of status of ‘I am ready’ (local maxima).

		Coordinates (mm)				
Region	Area	x	y	z	T value	P value
Frontal	L MFG	-42	51	16	5.73	< 0.001
	R MFG	44	47	11	5.36	< 0.0005
	L FEF	-34	-5	50	10.03	< 0.00005
	R FEF	32	-5	50	10.51	< 0.00001
	rCMA	-6	10	47	12	< 0.00001
	L PreCG	-42	-11	48	5.97	< 0.0001
	R PreCG	44	-13	45	8.1	< 0.0001
Parietal	L IPL	-50	-38	46	6.84	< 0.0005
	R IPL	53	-35	48	8.1	< 0.0001
	R IPS	34	-50	52	5.78	< 0.001

Note. Abbreviations: L, left; R, right; MFG, middle frontal gyrus; FEF, frontal eye field; rCMA, rostral cingulate motor area; PreCG, precentral gyrus; IPL, inferior parietal lobe, IPS, intraparietal sulcus; Coordinates: x, left/right; y, posterior/anterior; z, inferior/superior in the reference frame of the Talairach coordinates.

**Figure 4:**
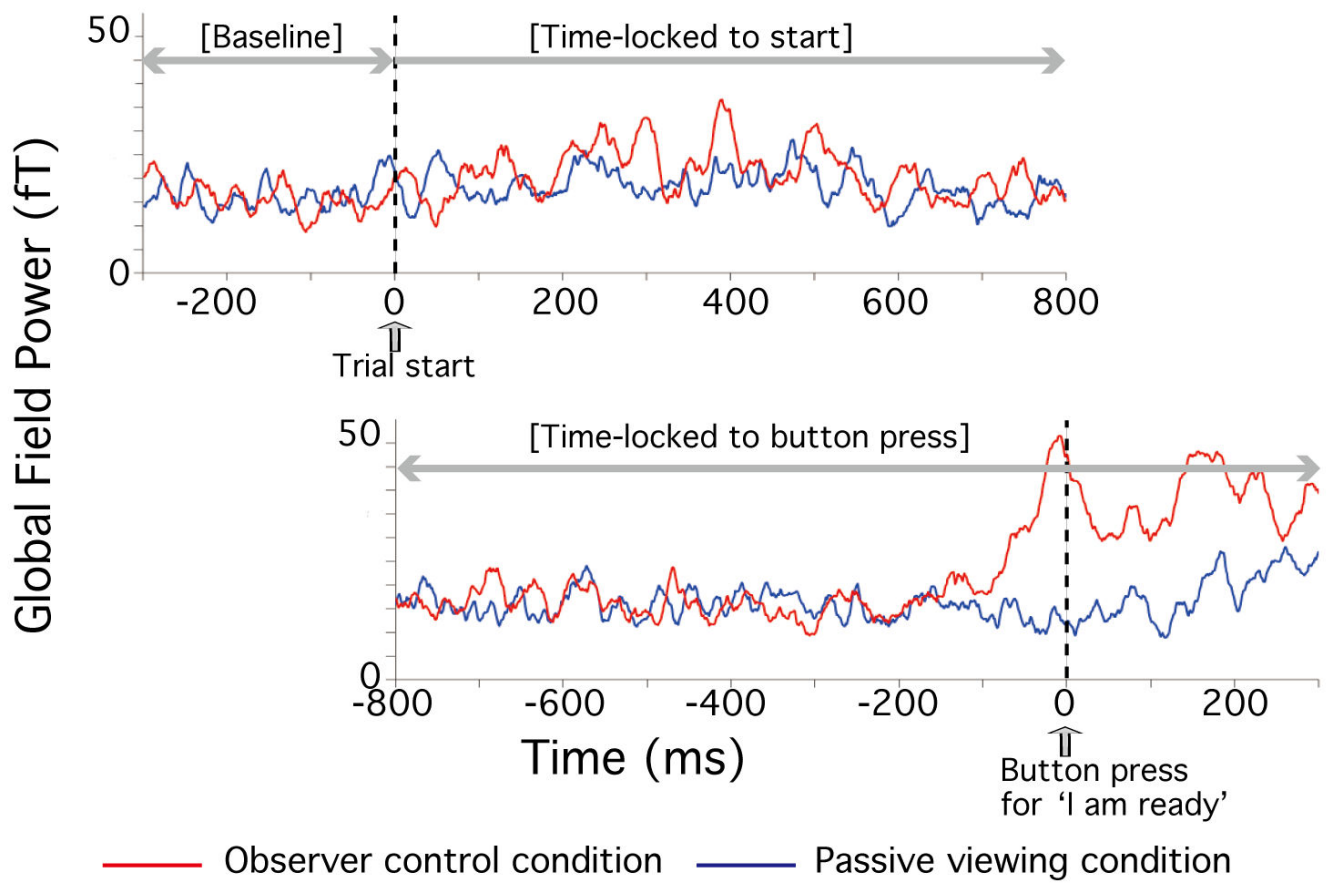
Examples of MEG data. The obtained data arranged: (1) time-locked to the start of the trial (Top panel), and (2) time-locked to the button press (Bottom panel). Examples of MEG data are shown for participant TT in both the observer-control condition (shown in red) and the passive control condition (shown in blue).

The time-locked-to-start data was 1100 msec long, consisting of 300 msec pre-trial data (i.e. baseline) plus 800 msec post-trial data (Top panel in Figure 4). This was the shortest duration required for any participant to signal (via a button press) they were ready for the stimulus to be presented. The time-locked-to-button-press data was also 1100 msec long, consisting of 800 msec pre-trial data plus 300 msec post-trial data (Bottom panel in Figure 4). For statistical analysis, the same baseline data was used as in the time-locked-to-start data.

### Neural oscillatory activity

To determine which, if any, cortical rhythms are affected during self-monitoring of attentional status, we performed time-frequency analyses by computing event-related spectral perturbation (ERSP) plots [17]. Perturbation plots were computed for each of the single estimated currents within each ROI for both conditions. The spectral power differences (in decibels) were referenced to a 300 msec baseline recording (see Methods). The power spectrum was determined using a Fast Fourier Transform (FFT), Hanning-windowed, designed to represent frequencies from 1.9 Hz to 50.8 Hz with 26 steps. The time dimension was evaluated over the 25 points for the data time-locked to the start of each trial, and 32 points for the data time-locked to the depression of the control-box button. At each time point, the FFT was estimated within a window of 256 msec. For each participant and for each ROI, we created mean ERSP plots for both the observer-control condition and the passive viewing condition. For each ROI, and for both the time-locked-to-trial-start data and the time-locked-to-button-press data, each pixel on the ERSP plot was submitted to statistical analysis (n = 10, paired-sample *t* test).

**Figure 5:**
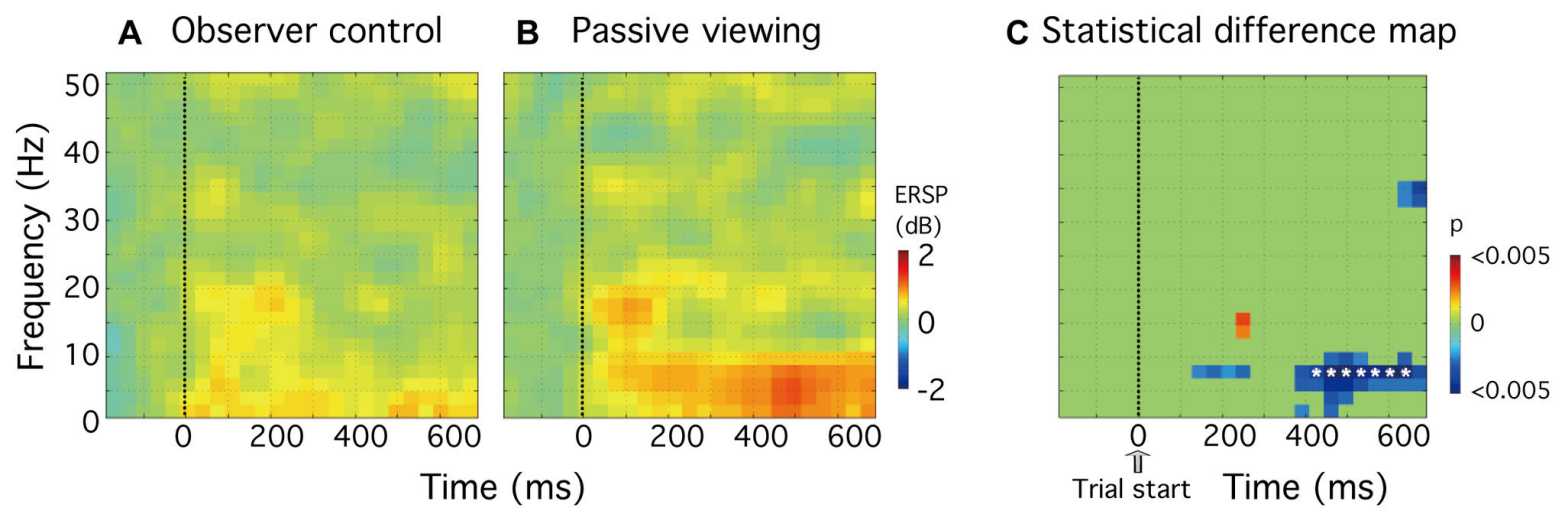
Group analysis (n=10) showing the self-monitoring related oscillatory activity for the time-locked-to-start data set in rCMA. **A** and **B**, The group-mean ERSP plots show power differences (in dB) referenced to a 300 msec baseline recording for (**A**) observer-control and (**B**) passive viewing conditions. **C**, Statistical significance map of the differences between the experimental conditions, as assessed using a paired-sample *t* test (n = 10, p < 0.05). Multiple comparisons were corrected for 7.8 Hz to 13.7 Hz, and results are indicated as white asterisks in Panel C (p < 0.05, FDR corrected; df = 9).

For the data time-locked to the start of each trial, significant differences between the observer-control and passive viewing conditions were only observed (p < 0.05) within rCMA (Figure 5). Figure 5B shows that within this area, in the passive viewing condition, an increase in spectral power (colored red) was evident for low frequencies (approximately 2-12 Hz), beginning some 70 msec after time zero (trial start). In the observer-control condition, however, no discernible increase in spectral power was evident within this range of frequencies (Figure 5A). Figure 5C shows a statistical significance map of the differences between the two experimental conditions (n = 10, paired-sample *t* test, p < 0.05). Because we have considerable published data showing that attentional modulation in humans affects the amplitude of cortical oscillations within the alpha frequency range [6,18,19], and because other studies suggest an important role for alpha-band activity in cognitive tasks [20], multiple comparisons were corrected for a prior defined frequency range 7.8 Hz to 13.7 Hz across the entire time period. Figure 5C shows that, from 430 msec to 650 msec post-trial start, alpha activity within rCMA was significantly less when participants self-monitored their attentional status (p < 0.05, FDR corrected; *df* = 9; white asterisks (t = 426, 461, 496, 531, 566, 602, 637 msec, f=7.8 Hz)).

**Figure 6:**
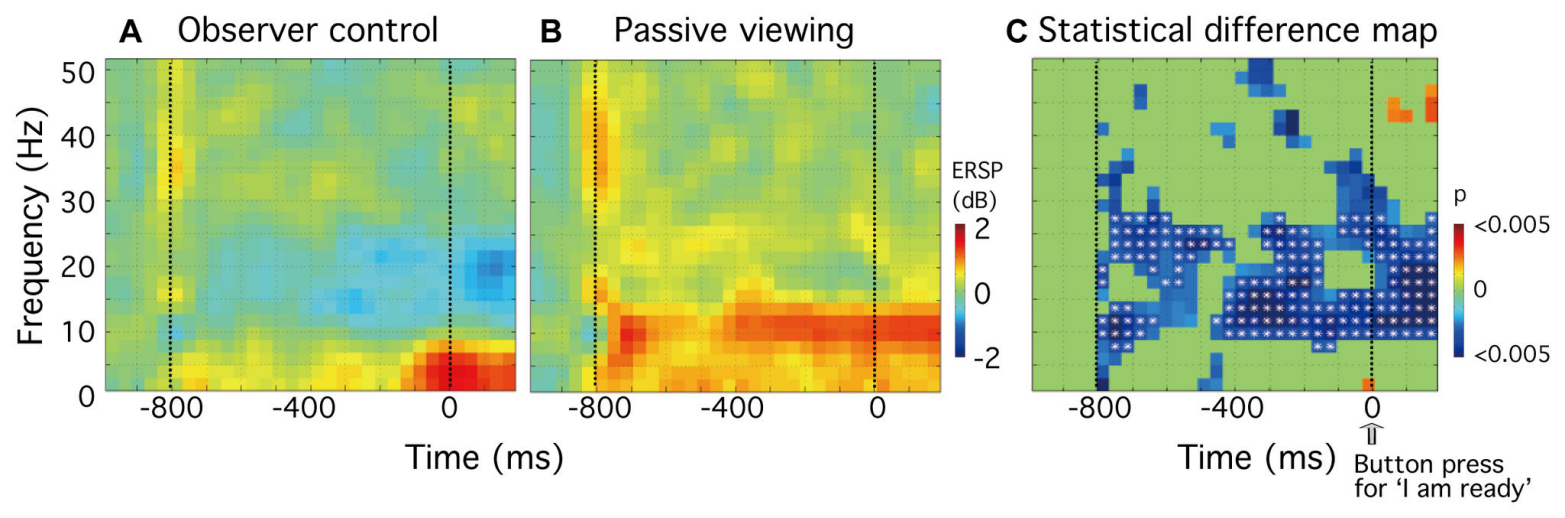
Group analysis (n=10) showing the differences between experimental conditions for the time-locked-to-button-press data set in left IPL. **A** and **B**, The group-mean ERSP plots show power differences (in dB) referenced to a 300 msec baseline recording for (**A**) observer-control and (**B**) passive viewing conditions. **C**, Statistical significance map of the differences between the experimental conditions, as assessed using a paired-sample *t* test (n = 10, p < 0.05). Multiple comparisons were corrected for 7.8 Hz to 27.3 Hz, and results are indicated as white asterisks in Panel C (p < 0.05, FDR corrected; df = 9).

For data time-locked to depression of the control-box button, significant differences were observed between experimental conditions (p < 0.05) in the left inferior parietal lobe (IPL) (Figure 6). Within this region, in the 800 msec prior to depression of the button to initiate stimulus onset, an increase in spectral power (colored red) was evident for low frequencies (approximately 2-14 Hz) in the passive condition (Figure 6B). In this same time period a decrease in power (coloured blue) was evident for middle frequencies (approximately 10-23 Hz) in the observer-control condition (Figure 6A). Figure 6C shows a statistical significance map of the differences between experimental conditions (n = 10, paired-sample *t* test, p < 0.05). Since modulations of the Mu (8-13 Hz) and beta (14-25 Hz) rhythm have been reported for motor preparation [21-23], multiple comparisons were corrected for a prior defined frequency band 7.8 Hz to 27.3 Hz. Note that significant suppression of alpha and beta rhythms (approximately 10 to 25 Hz) occurred during the 800 msec before the control-box button was depressed, and for at least 200 msec after the button press (Figure 6C; white asterisks).

### Relationship between ERSP and task performance within rCMA

**Figure 7:**
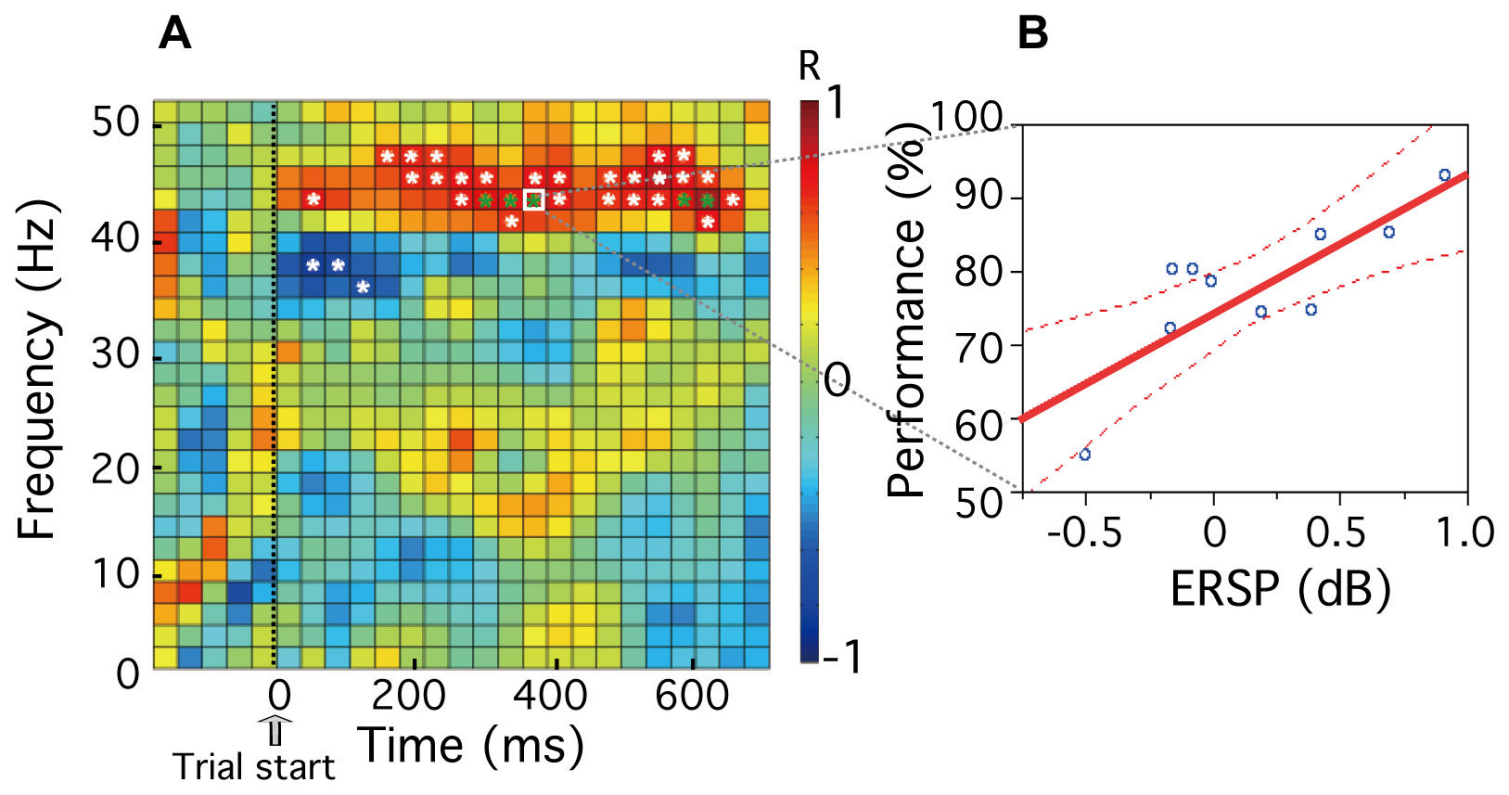
Relationship between event-related spectral perturbation (ERSP) and task performance within rCMA. **A**, Correlation between ERSP values and task performance. White asterisks indicate time/frequency points where p < 0.05, mean r = 0.71. Multiple comparisons were corrected for the entire time period with a frequency of 43Hz, and results are indicated as green asterisks (p < 0.05, FDR corrected; *df* = 9, mean r = 0.79). **B**, Example of correlation between ERSP and task performance for the time/frequency point indicated by a white box in Panel A (t=355 msec, f=43.0 Hz). The solid red line shows linear regression fits to the data, and the broken lines show the 95% confidence interval.

Using the data for all observers (n=10), we tested for correlations between task performance in the observer-control condition and ERSP values within the rCMA. For each participant, a correlation coefficient between performance (ACC) and ERSP values at each frequency and at each time point (i.e. each cell in Figure 7A) was determined (see Figure 7B). Note that no correlation between performance and ERSP was evident for any cells within the alpha band (near 10Hz, Figure 7A). However, a strong positive relationship between gamma activity and task performance was evident within several cells (greater than 40 Hz; colored red in Figure 7A). Sustained correlations (p < 0.05, uncorrected) were found within the frequency range 41 Hz to 47 Hz from 39 msec to 637 msec. Because positive correlations between gamma power changes and behavioral performance have been previously demonstrated [24,25], multiple comparisons were corrected for a prior defined frequency range 41 Hz to 47 Hz across the entire time period. Five significant points (t = 285, 320, 355, 566, 602 msec, f = 43.0 Hz), indicated by the green asterisks in Figure 7A, were revealed (p < 0.05, FDR corrected). The correlation between ERSP values and task performance at t=355 msec and f=43.0 Hz is shown in Figure 7B (r=0.807), where the broken lines are the 95% confidence interval.

### Relationship between alpha and gamma modulations

**Figure 8:**
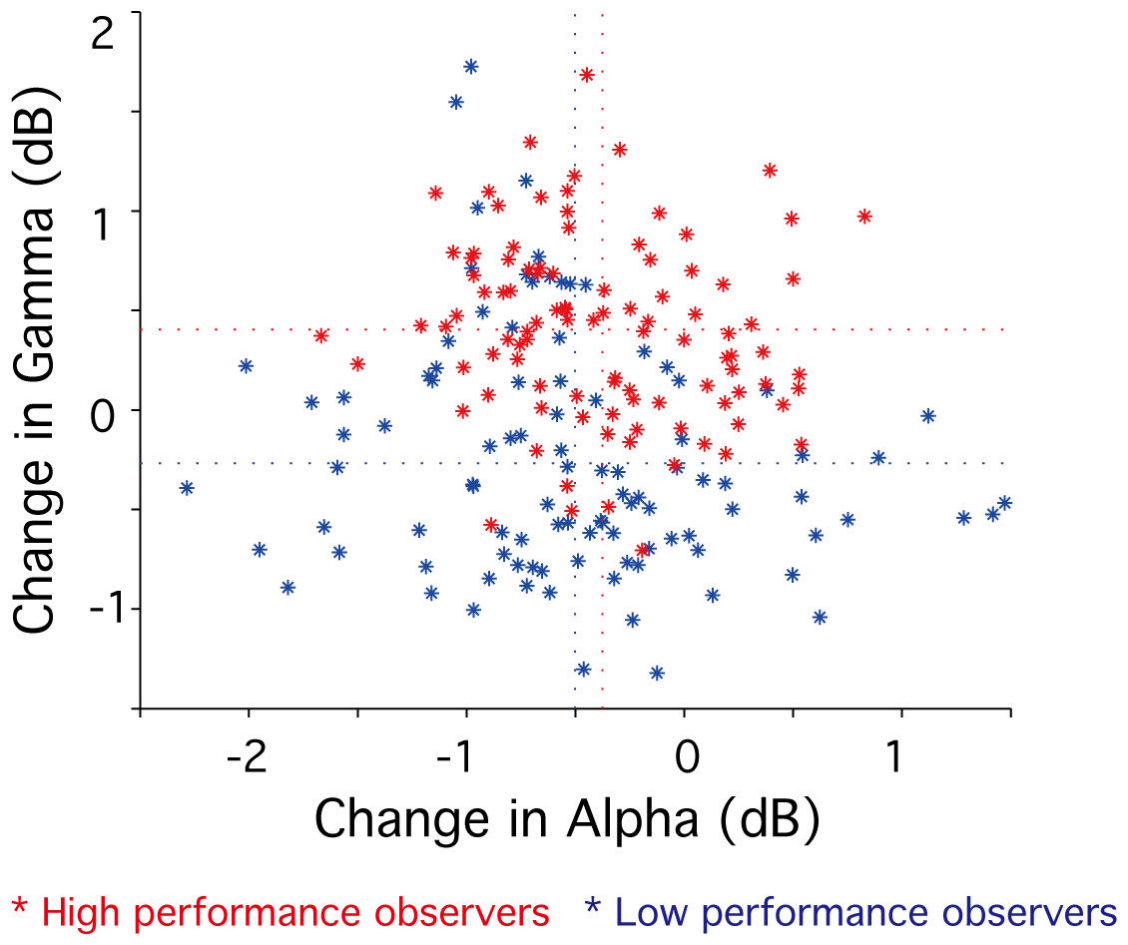
Relationship between alpha and gamma modulations. Differences of ERSP values between experimental conditions in gamma (43.0 Hz) frequency are plotted against differences of ERSP values in alpha (7.8 Hz). Data are shown for high performance observers (n=5, ranges 54.9-78.6%, mean 71.0%, shown in red) and low performance observers (n=5, ranges 80.1-93.0%, mean 84.8% shown in blue). Horizontal dotted lines indicate means of gamma changes and vertical dotted lines indicate means of alpha changes in each experimental condition.

To clarify the relationship between alpha and gamma modulations, we compared power changes between the observer-control and the passive viewing conditions (in dB) for 7.8 Hz (alpha) and 43.0 Hz (gamma) frequencies at each time point (t = 4-672 msec, 20 time points), for data time-locked to the trial start. Figure 8 shows, for each observer, gamma changes plotted against alpha changes. Observers were divided into high and low performance groups based on their psychophysical task performance measure. A performance measure of < 80% was defined as low (n = 5, performance: ranges 54.9-78.6%, mean 71.0%, data are shown in blue); and a measure of > 80% was defined as high (n = 5, performance: ranges 80.1-93.0%, mean 84.8%, data are shown in red). Note that no significant correlation between alpha and gamma changes was evident for either low- or high-performance observers (high performance group, r = -0.15, p > 0.05; for low performance group, r = -0.19, p > 0.05). Note also that there was no significant difference in alpha changes between the groups (p > 0.2, mean of alpha changes [high, low] = [-0.376, -0.502]). However, there was significant difference in gamma changes between the groups (p < 0.001, mean of gamma changes [high, low] = [0.404, -0.267]): gamma was significantly greater in the high performance group compared with the low performance group.

### Neural activity in rCMA: data time-locked to orientation judgment

**Figure 9:**
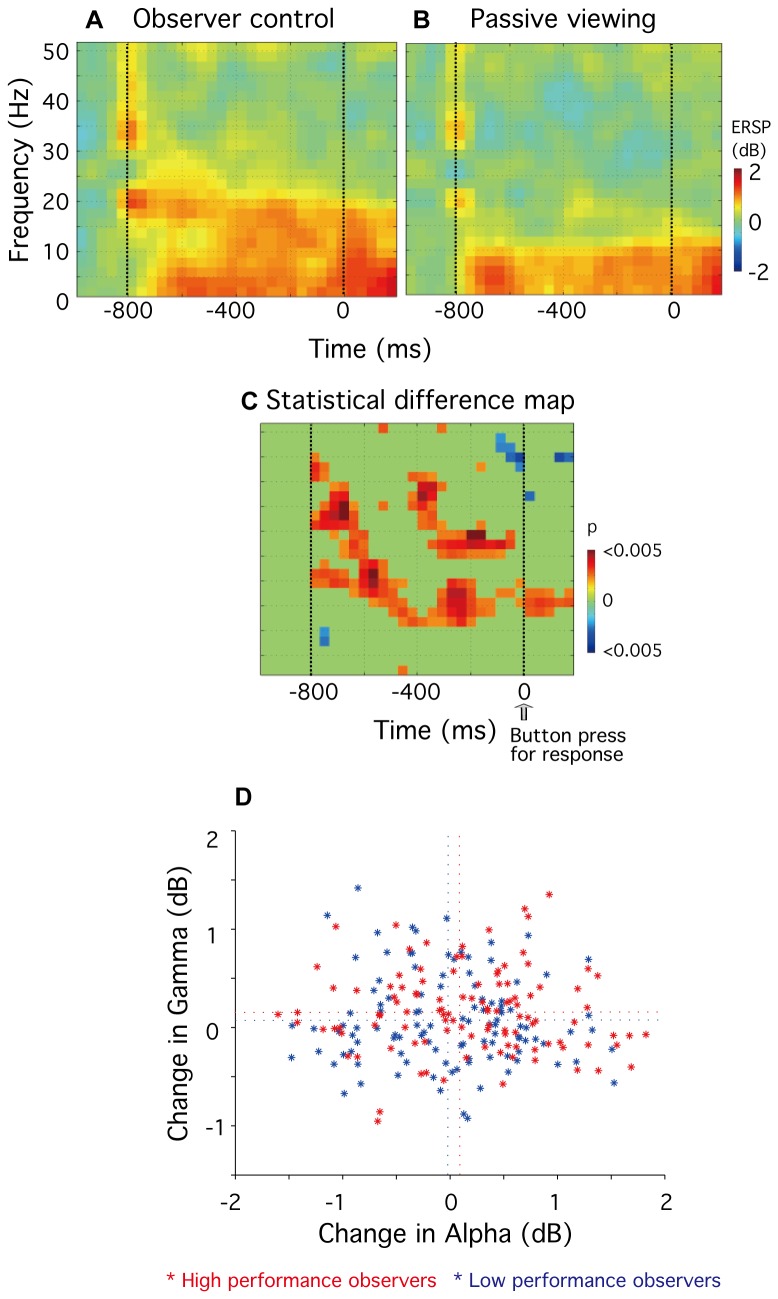
Group analysis (n=10) showing neural activity for data time-locked to the orientation judgment button press in rCMA. **A** and **B**, The group-mean ERSP plots show power differences (in dB) referenced to a 300 msec baseline recording for (**A**) observer-control and (**B**) passive viewing conditions. **C**, Statistical significance map of the differences between the experimental conditions, as assessed using a paired-sample *t* test (n = 10, p < 0.05 uncorrected). **D**, Relationship between alpha and gamma modulations. Differences of ERSP values between experimental conditions in gamma (43.0 Hz) are plotted against differences of ERSP values in alpha (7.8 Hz) for 22 time points (-787 to -13 msec before the button press). Data are shown for high performance observers (n=5, shown in red) and low performance observers (n=5, shown in blue). Horizontal dotted lines indicate means of gamma changes and vertical dotted lines indicate means of alpha changes in each experimental condition.

For data time-locked to the start of each trial, significant differences between the observer-control and passive viewing conditions were observed within the area rCMA: alpha activity within rCMA was significantly less when participants self-monitored their attentional status (see Figure 5), and there was a significant positive relationship between gamma activity and task performance (see Figures 7 and 8). To provide further evidence that these observed effects within rCMA were related to self-monitoring of attention, and not simply to general attentional processes and/or motor-related activity, we repeated the analyses for data time-locked to the orientation judgment button press. The results are shown in Figure 9. Note that in both the observer control (9A) and passive viewing (9B) conditions, an increase in spectral power (colored red) was evident across a broad range low frequencies, beginning around 800 msec before the button press. Figure 9C shows a statistical significance map of the differences between the two experimental conditions (n = 10, paired-sample *t* test, p < 0.05). Note that, for data time-locked to the button press, there was no evidence for a reduction in alpha (7.8 Hz) within rCMA (compare Figure 9C with Figure 5C). We also assessed the relationship between alpha and gamma modulations for both high- and low-performance participants. Unlike the results for data time-locked to the trial start (Figure 8), there was no evidence for increased gamma (43.0 Hz) activity with high performance participants for data time-locked to the orientation judgment button press (compare Figure 8 with Figure 9D). Note also there was no significant difference in either alpha or gamma changes between the two performance groups (p > 0.05, mean of alpha changes [high, low] = [0.170, -0.025]; p > 0.05, mean of gamma changes [high, low] = [0.165, 0.080]). In brief, we could find no evidence for decreased alpha or increased gamma rhythms within rCMA for data time-locked to the orientation judgment button press.

## Discussion

Using neuroimaging techniques (MEG and fMRI) in combination with psychophysical measures of visual performance, we sought to determine whether oscillatory brain rhythms play a role in the neural processes involved in self-monitoring attentional status. Behaviorally, we have shown previously that orientation discrimination is significantly improved when observers self-monitor their attentional status for the purpose of maximising performance [10]. We used the same orientation discrimination task in this study and found – using fMRI – a number of candidate regions within the frontal and parietal cortex that may be associated with self-monitoring (Figure 3, Table 1). Computing event-related spectral perturbation (ERSP) plots for representative current dipoles within each identified region we went on to show that, for data time locked to the beginning of each trial, self-monitoring is associated with a sustained power decrease of alpha activity (7-13Hz) within the rostral cingulate motor area (rCMA, Figure 5C). Although we did not find a significant correlation between alpha power and task performance within this area (Figure 7), we did find a significant positive correlation between gamma-band power (41-47 Hz) and performance (Figure 7). While all participants demonstrated decreased alpha power, only high-performance participants showed increased gamma power in rCMA (Figure 8).

Different effects of attention on alpha and gamma rhythms within a given neural area were recently reported by Buffalo et al. [7]. They showed that gamma was dominant within the superficial layers areas V2 and V4 in primate while alpha was dominant within the deep layers, and that attention affected those dominant frequencies in opposite ways: attention enhanced gamma synchrony but reduced alpha synchrony. In consideration of the known anatomical projection targets of different cell layers within areas V2 and V4, Buffalo et al. [7] concluded that the changes in gamma synchrony are most likely to affect higher cortical areas, while the changes in alpha are most likely to affect subcortical structures and perhaps V1.

The qualitative differences we observed between alpha and gamma within rCMA presumably reflect their different neural roles in the self-monitoring of attentional status. It remains an open question, however, as to what those roles might be. Indeed, although oscillatory brain rhythms have been associated with a number of cognitive [26] and sensori-motor actions [27], their functional significance remains unclear. Certainly, alpha desynchronization has long been associated with attentional processes [5,6,18,19,28-30], with the general consensus being that spatially localized reductions in alpha reflect areas of heightened neural activity [27,31]. Other research suggests that changes in alpha rhythms may also form part of interareal feedback communication networks for attentional control [6,32], and more recent studies suggest that local changes in alpha may reflect engagement or disengagement within local brain regions [33]. In contrast to alpha coherence, gamma coherence is known to be enhanced by attention [34], a change which may serve to enhance the impact of attended sensory signals on downstream neurons [7,35,36].

Using functional connectivity magnetic resonance imaging (fcMRI), Habas [37] reported that both rostral and caudal parts of the cingulate motor areas in human are functionally connected to the dorsolateral prefrontal, rostral cingulate, rostral insular, sensorimotor cortices, and rostral striatum. Specifically, the rCMA displays more widespread prefrontal and orbitofrontal, premotor, and medial parietal cortical connections. These areas are variously involved in the neural mechanisms of planning [38], imagery movement [39], episodic memory retrieval [40], self judgments [41], self-awareness [42], and attention to self [43].

Primate studies suggest a role for rCMA in motor execution [44], visuomotor transformation [45], voluntary movement selection [46], and the planning and execution of movements [47]. Human studies provide general support for these findings, suggesting the rCMA may help determine the speed of reaction times [48], and is involved in conflict monitoring [49] and attention-demanding cognitive tasks [50].

Habas (2010) [37] suggested that the cingulate motor areas may constitute an interface between sensorimotor, limbic and executive systems. This fits well with the reported roles of these areas from both animal and human studies, and with the results of our current neuroimaging study. Activation of rCMA, as defined by the changes we observed in alpha- and gamma-band power, is entirely consistent with the required actions of our observers, i.e. self-monitoring of their attentional status, responding when it was maximal with an appropriate motor movement for stimulus presentation and discrimination. We conclude that, in addition to its reported roles, rCMA in humans may help evaluate competing internal signals for the purpose of maximising behavioural performance. In this respect we speculate that alpha and gamma brain rhythms may act in feedback and feedforward processes, respectively. Specifically, we suggest that alpha suppression reflects a strengthening of top-down interareal connections, and that gamma enhancement plays an important feedforward role in guiding visuomotor behavior.

A different picture emerged for data time-locked to depression of the control-box button. In this case, a significant suppression of power within the 10-25 Hz frequency band was evident within the left inferior parietal lobe (IPL) (Figure 6C). This is consistent with previous studies that report a suppression of Mu (8-13 Hz) and beta activity (14-30 Hz) within the contra-lateral parietal area before either real [51] or imaginary movement [52]. It is also consistent with physiological studies reporting that neural activity within the parietal cortex may reflect the integration of sensory signals relevant to a decision for movement [53,54]. Given these findings, we assume the suppression of 10-25 Hz activity we observed within the IPL reflects preparatory motor activity.
